# Motion compensated reconstruction improves image quality and interpretability of dual-layer coronary CT angiography

**DOI:** 10.1007/s00330-025-11946-x

**Published:** 2025-09-03

**Authors:** Philip M. Tetteroo, Niels R. van der Werf, Isabelle Bax, Mani Vembar, Tim Leiner, Pim A. de Jong, Birgitta K. Velthuis, Dominika Suchá

**Affiliations:** 1 Department of Radiology & Nuclear Medicine, University Medical Center Utrecht, Utrecht, The Netherlands; 2 Philips, Best, The Netherlands; 3 CT Clinical Science, Philips Healthcare, Cleveland, OH USA; 4 Department of Radiology, Mayo Clinic, Rochester, MN USA

**Keywords:** Coronary CT angiography, Coronary vessels, Motion artifact, Motion correction, Image quality

## Abstract

**Objectives:**

Reducing motion artifacts in coronary computed tomography angiography (CCTA) is essential for accurate coronary artery disease assessment. We evaluated the clinical performance of a motion-compensated reconstruction (MCR) using subjective image quality (SIQ) and interpretability of CCTA at varying heart rates (HR).

**Materials and methods:**

We retrospectively identified 150 patients, grouped by HR (≤ 60, 60–69, ≥ 70 bpm, *n* = 50 each), referred for prospective ECG-gated CCTA on a spectral dual-layer CT. Two blinded observers independently assessed SIQ on a per-segment (≥ 1.5 mm) and per-patient level using a 4-point Likert scale in 18 coronary segments (78% RR-interval). Sufficient diagnostic interpretability was defined as SIQ ≥ 2. Per-vessel scores were calculated excluding side branch segments. Per-segment SIQ interobserver agreement was assessed using Cohen’s Weighted Kappa. Between MCR and standard reconstruction (SR) at 78% RR-interval, SIQ was compared with Wilcoxon signed-rank tests and diagnostic interpretability and HR-categories using McNemar tests.

**Results:**

Mean age was 57 (50–64) years, with 50% men, and 1970 included segments. Interobserver agreement was 0.80 for SR and 0.77 for MCR. Positive trends of improved SIQ were seen across all HR-categories and levels, with significant improvements in all but ≥ 70 bpm on a patient level (*p* = 0.22). Likewise, positive trends were seen for diagnostic interpretability across all levels and HR-categories with significant improvements at the per-segment level for HR > 60 bpm and per-patient level for 61–69 bpm.

**Conclusion:**

Compared to the standard reconstruction at 78% RR-interval, MCR significantly improves SIQ and diagnostic interpretability in patients referred for CCTA in most HRs and major vessels (≥ 1.5 mm).

**Key Points:**

***Question*** *Motion artifacts hinder the assessment of coronary arteries on coronary CT angiography (CCTA), leading to more non-diagnostic segments or scans.*

***Findings*** *Compared to the standard reconstruction 78% RR-interval, motion compensated reconstruction (MCR) significantly improves subjective image quality (SIQ) and diagnostic interpretability across heart rate categories.*

***Clinical relevance*** *By integrating multi-phase data into an optimized single-phase reconstruction with improved SIQ and diagnostic interpretability, MCR may reduce the need for multi-phase assessments when the target phase is non-diagnostic.*

**Graphical Abstract:**

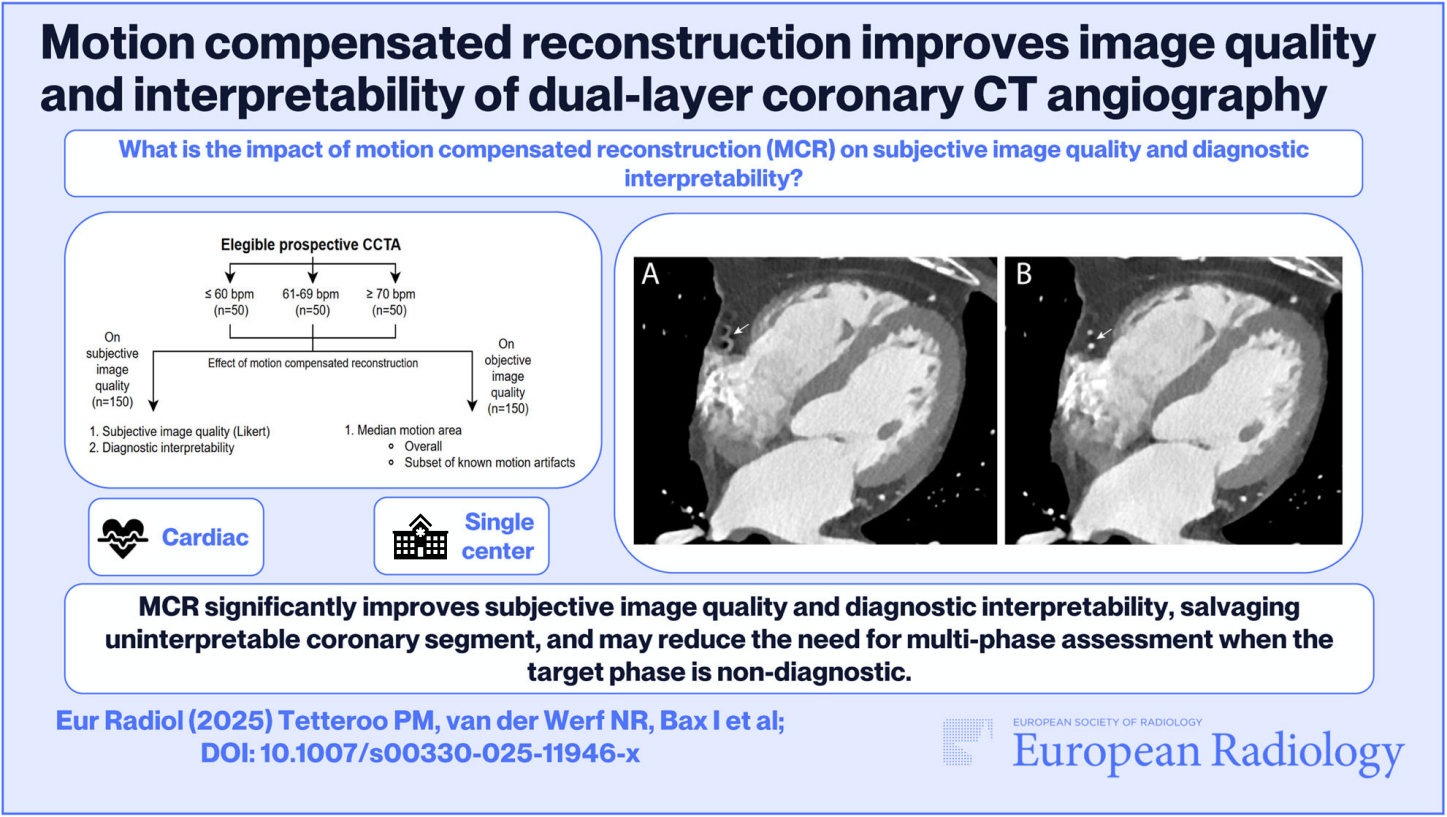

## Introduction

Coronary computed tomography angiography (CCTA) is a widely used non-invasive imaging modality for assessing coronary artery disease (CAD) [1]. It is the primary diagnostic tool for patients with stable chest pain and intermediate to high cardiovascular risk [2]. CCTA may also be considered when functional tests are inconclusive, or in select acute chest pain patients with atypical troponin and electrocardiography (ECG) results, as per European Society of Cardiology (ESC) guidelines [3].

CCTA has grown significantly for diagnosing and managing CAD [4]. Clinical indications are increasing beyond diagnostic purposes to include therapy planning and preventive medicine [5–7]. This growth in use and scope is reflected in new Coronary Artery Disease-Reporting and Data System (CAD-RADS) 2.0 guidelines, which recommend more comprehensive plaque assessment, and in the rising interest in artificial intelligence (AI) tools for (semi)automated plaque detection and characterization [5, 8]. High-quality CCTA acquisitions are important, as they may reduce unnecessary invasive coronary angiography, enable accurate diagnostic and treatment decision-making, and facilitate efficient use of automation and advanced deep learning tools [6, 9–11].

However, challenges persist in managing cardiac and coronary artery motion during CCTA. The right coronary artery (RCA) and circumflex artery (Cx) are prone to motion artifacts due to their anatomical location and orientation [3, 8, 12–14]. While technical developments and patient preparation with beta-blockers can mitigate motion, limitations remain. Up to 30% of patients may not achieve the targeted HR, leading to 10% of CCTA scans being performed above 70 beats per minute (bpm) [15, 16]. Thus, there is a clear need for further improvements to image quality (IQ), thereby minimizing non-diagnostic scans.

Advanced post-processing approaches have been developed to correct for cardiac motion to further improve IQ [17–23]. In our study, we use a vendor-specific motion-compensated reconstruction (MCR) (“Precise Cardiac,” Philips Healthcare) incorporating all the steps as part of the reconstruction pipeline, leveraging data from adjacent cardiac phases within a single cardiac cycle to generate motion-corrected images [24–28]. Our dynamic phantom study using this approach demonstrated promising results by reducing the area of motion artifacts by up to a factor of 11 [25]. However, clinical evaluation is essential to validate these findings. This patient study aims to compare the clinical performance of this vendor-specific MCR with standard reconstructions (SRs) on subjective and objective IQ and diagnostic CCTA interpretability at varying HR categories and plaque load.

## Methods

### Study subjects

Informed consent was waived by the local Medical Research Ethics Committee (decision number: 22-095). We retrospectively identified all adult patients who underwent CCTA on a spectral dual-layer computed tomography (DLCT; CT 7500, Philips Healthcare) at the University Medical Center Utrecht between April 2021 and April 2022 (Appendix 1). Consecutive patients with a regular sinus rhythm on ECG prior to the acquisition were selected who underwent (A) CCTA according to our prospective ECG-triggered acquisition protocol, including MCR, and (B) matched a HR category as described below. The average HR prior to CCTA acquisitions was routinely stored in the DICOM data elements and patients were divided equally into three HR categories: ≤ 60, 60–69, and ≥ 70 bpm, with 50 patients per category. Exclusion criteria were (1) severe step artifacts causing ≥ 1 missing segment (*n* = 2) and (2) severe motion artifacts in all segments regardless of reconstruction (*n* = 2). Patient baseline characteristics were obtained from medical records and scan parameters from DICOM data elements, Table 1.

**Table 1:** Coronary CTA acquisition and reconstruction parameters

Parameter	Setting
Acquisition	
Scan mode	Sequential
Tube voltage (kVp)	120
Tube current time product (Ref. mAs)	176
Dose right index	22
Phase tolerance (%)	2
Collimation (mm)	128 × 0.625
Field of View (mm)	220^a^
Rotation time (s)	0.27
Slice thickness/increment (mm)	0.9/0.45
Matrix size (pixels)	512 × 512
Reconstruction	
Reconstruction kernel and setting	IMR 1/cardiac routine

*kVp* kilovolt peak, *mAs* milliampere-seconds, *IMR* iterative model reconstruction

^a^ Adjusted per individual patient

### Patient preparation

Depending on the patient’s HR and the cardiologist’s assessment, patients with HR > 60 bpm were instructed to take 50 mg oral beta blocker (Metoprolol tartrate) two hours before the coronary CT, with some receiving an additional dose the day before the procedure. If high HR (≥ 65 bpm) persisted, additional intravenous beta-blockers (Metoprolol (Seloken) 5–20 mg) were administered directly preceding the CCTA until, ideally, an average HR of < 65 bpm was reached. Additionally, two doses of sublingual nitroglycerin were administered before CCTA to patients with systolic blood pressures ≥ 100 mmHg and no aortic valve stenosis.

### Acquisition protocol

The CT protocol included a calcium score acquisition followed by a prospective ECG-triggered CCTA. The CCTA imaged the heart at 78% RR-interval diastolic phase with a 2% phase tolerance, resulting in an x-ray over over 360 degrees and enabling the extraction of relevant information over this temporal range for the use of MCR. A triphasic contrast injection protocol (70 or 80 mL of 300 mg I/mL Iopromide, Bayer Inc.) was used, followed by a 1:1 ratio mixture of contrast medium and saline (50 or 67 mL) and lastly a saline chase (30 or 40 mL), all injected at 6 or 6.7 mL/s flowrates based on bodyweight (< 80 or ≥ 80 kilogram, respectively). Acquisition was triggered by bolus tracking in the descending aorta (150 Hounsfield Units (HU) threshold) and a 7-second post-threshold delay. Acquisition parameters included a tube voltage of 120 kVp, tube current of 176 ref. mAs, DoseRight Index (DRI) of 22, and rotation time of 0.27 seconds (Table 1). All CCTA images were reconstructed with iterative model reconstruction (IMR) level 1 and a cardiac routine setting.

### Motion correction methodology

Two datasets (SR and MCR) were reconstructed for each scan, targeting the central cardiac phase of interest (78%), and included for the IQ assessment. While both are half-scan reconstructions, the vendor-specific MCR approach used in this study comprises additional steps wherein tubular structures are first detected over the search region around the targeted phase. This is followed by elastic registration from which the motion pattern is modeled and used as part of back projection [24–28]. Further technical details are explained in Schirra et al [28]. MCR has been fully automated and integrated into the reconstruction process.

### Image quality

Objective IQ (OIQ) was assessed using the lumen area, assuming that an increased lumen area and motion artifact are correlated [25]. For this purpose, a single cross-sectional slice of mid-segment 2 of the RCA was manually selected in all patients. As segment 2 experiences the most motion, it is likely the most appropriate segment to evaluate the impact of MCR [29]. For the analysis of both the SR and MCR, a previously used in-house developed Python script was applied [25]. A 120 HU threshold isolated the lumen, including motion artifacts from surrounding tissue. A connected component analysis was subsequently used to fully automatically quantify the total vessel area on the selected slice.

**Fig. 1:**
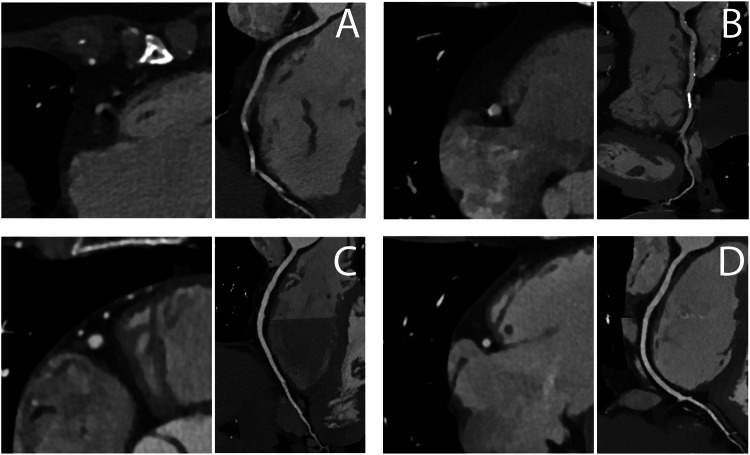
Example of Likert scores 1–4. Clinical examples of the Likert scores, presented for the right coronary artery in segment 2 or 3 (standard reconstruction at 78% RR-interval cardiac phase). The top-left image (**A**) shows a Likert score of 1: non-diagnostic, top-right (**B**) a Likert score of 2: suboptimal/reduced but interpretable for clinical decision-making, bottom-left (**C**) a Likert score of 3: good with mild motion or other artifacts (step artifact), and bottom-right (**D**) a Likert score of 4: excellent with minimal to no motion-related artifacts and excellent anatomic detail for stenosis assessment and plaque evaluation

Subjective IQ (SIQ) was independently assessed by two cardiovascular radiologists (D.S. and B.V.), each with > 10 years of experience in cardiovascular CT. SIQ was assessed per-segment (18-segment SCCT model) and per-patient level using a 4-point Likert scale: 1: non-diagnostic SIQ, 2: suboptimal (reduced) SIQ but interpretable for clinical decision-making, 3: good SIQ with only mild motion or other artifacts, 4: excellent SIQ with minimal or no motion-related artifacts and excellent anatomic detail for stenosis assessment and plaque evaluation (Fig. 1). Scores ≥ 2 were considered diagnostically interpretable. Non-interpretable segments subsequently classified the corresponding vessel and patient as non-diagnostic too, with reasons reported as: noise (1.1); low contrast (1.2); motion artifact (1.3); metal/clip artifact (1.4); other (1.5). Segments too small (< 1.5 mm) for clinical decision-making were excluded as per CAD-RADS guidelines [8]. For a per-vessel level analysis, scores were calculated based on the main segments: 1–3 (RCA), 5 (left main), 6–8 (left anterior descending artery), and 11 and 13 (Cx) to prevent a disproportionate impact of often smaller vessels on the results. We acknowledge that this approach excludes some potentially larger branches, such as the diagonal, septal, or intermedius branches. Both datasets were scored on a dedicated workstation (PACS, Sectra) with at least a two-week interval to reduce recall bias. Observers were blinded to the reconstruction method and to clinical information. CCTA images were presented in the standard transverse plane. Multiplanar reformations, maximal intensity projection reconstructions, and window-level settings were at the reader’s discretion. Discrepancies in anatomical coronary segment interpretation were resolved through consensus.

### Statistical analysis

Patient characteristics and continuous data are presented as mean ± standard deviation (SD) or median (25–75% quartiles), depending on data distribution. Categorical data are presented as a number (percentage). Normality of data distribution was assessed using the Shapiro–Wilk test and visual inspection. Independent-samples *t*-tests or Mann–Whitney *U*-tests were performed for normally and non-normally distributed continuous data, respectively, and Chi^2^ or Fisher’s exact tests for categorial data. The Wilcoxon signed-rank test assessed differences between SR and MCR for the OIQ area. Segment-level SIQ interobserver agreement was assessed using Cohen’s Weighted Kappa. Segments with scores of 1.1 (noise), 1.4 (metal/clip artifact), or 1.5 (other) were excluded from further analyses. SIQ differences between SR and MCR were compared with Wilcoxon signed-rank tests and diagnostic interpretability using McNemar tests for all patients (combined HR) and per HR category at all assessed levels. The effect and potential limitations of MCR on individual segments were further evaluated by analyzing the total number of non-diagnostic scores for each segment at ≥ 70 bpm, as this heart rate category is most susceptible to motion artifacts. Furthermore, to assess the incremental value of MCR when all reconstructed phases (76%, 78%, 80%, and MCR) are available, a single blinded observer systematically ranked all four reconstructions according to two key parameters: (1) overall image quality and (2) diagnostic interpretability, with tied rankings permitted when phases demonstrated comparable performance. The data were analyzed using IBM SPSS Statistics (Version 29.01) and Python version 3.8.

## Results

### Patient characteristics and included segments

A total of 150 patients were included (50 per HR category). Median age was 57 (50–64) years, and 75 (50%) were men. Demographic data are summarized in Table 2. In 150 patients, 730/2700 (27%) standard coronary segments were non-existent/missing due to anatomical variation, too small (< 1.5 mm) for clinical assessment (mainly segments 15, 17 and 18), or excluded because of noise, metal/clip artifacts, or other reasons as described previously. Hence, 1970 (73.0%) segments were included for analyses. Four patients were excluded from the multi-phase comparison due to missing additional phases.

**Table 2:** Baseline characteristics

Patient characteristics	All bpm	≤ 60 bpm	61–69 bpm	≥ 70 bpm
Patients	150 (100%)	50 (33%)	50 (33%)	50 (33%)
Age	57 (50–64)	61 (53–68)	59 (52–65)	52 (46–60)
Male	75 (50%)	24 (48%)	24 (48%)	27 (54%)^a^
Height (cm)	175 (±10.8)	172.5 (169–185)	176 (169–185)	175 (±11.3)
Weight (kg)	82.2 (±17.6)	77.5 (69–88)	81 (70–97)	85 (±22.4)
BMI	26.7 (±4.5)	26.1 (±3.7)	26.5 (24–29)	27.4 (±5.8)^a^
Heart rate (bpm)	64 (58–71)	57 (±7.3)	64 (±2.5)^a^	74 (±5.0)^a^
Smoker				
Never	56 (55%)	17 (54%)	22 (58%)	17 (53%)
Past	24 (24%)	10 (32%)	9 (24%)	5 (16%)
Current	21 (21%)	4 (13%)	7 (18%)	10 (31%)
Hypertension	47/146 (32%)	12/49 (24%)	17/48 (35%)	18/49 (37%)
Hyperlipaemia	36/144 (25%)	14/49 (29%)	12/47 (26%)	10/48 (21%)
Diabetes type 2	10/144 (7%)	2/48 (4%)	3/47 (6%)	5/49 (10%)
Family history	77/124 (62%)	21/41 (51%)	31/43 (72%)	24/40 (60%)
OSAS	4/144 (3%)	0/49	2/46 (4%)	2/49 (4%)
Alcohol	40/92 (43%)	14/31 (45%)	14/36 (39%)	12/25 (48%)
Sport (hours/week)	0.9 (±2.1)	1.2 (±2.7)	0.7 (±1.5)	0.8 (±1.7)

All values are presented as median (interquartile range (IQR)), mean (standard deviation (SD)), *n* (%), or hours

*bpm* beats per minute, *BMI* body mass index, *OSAS* obstructive sleep apnea syndrome

^a^ Significant *p*-values compared to **≤** 60 bpm

### Objective image quality

**Fig. 2:**
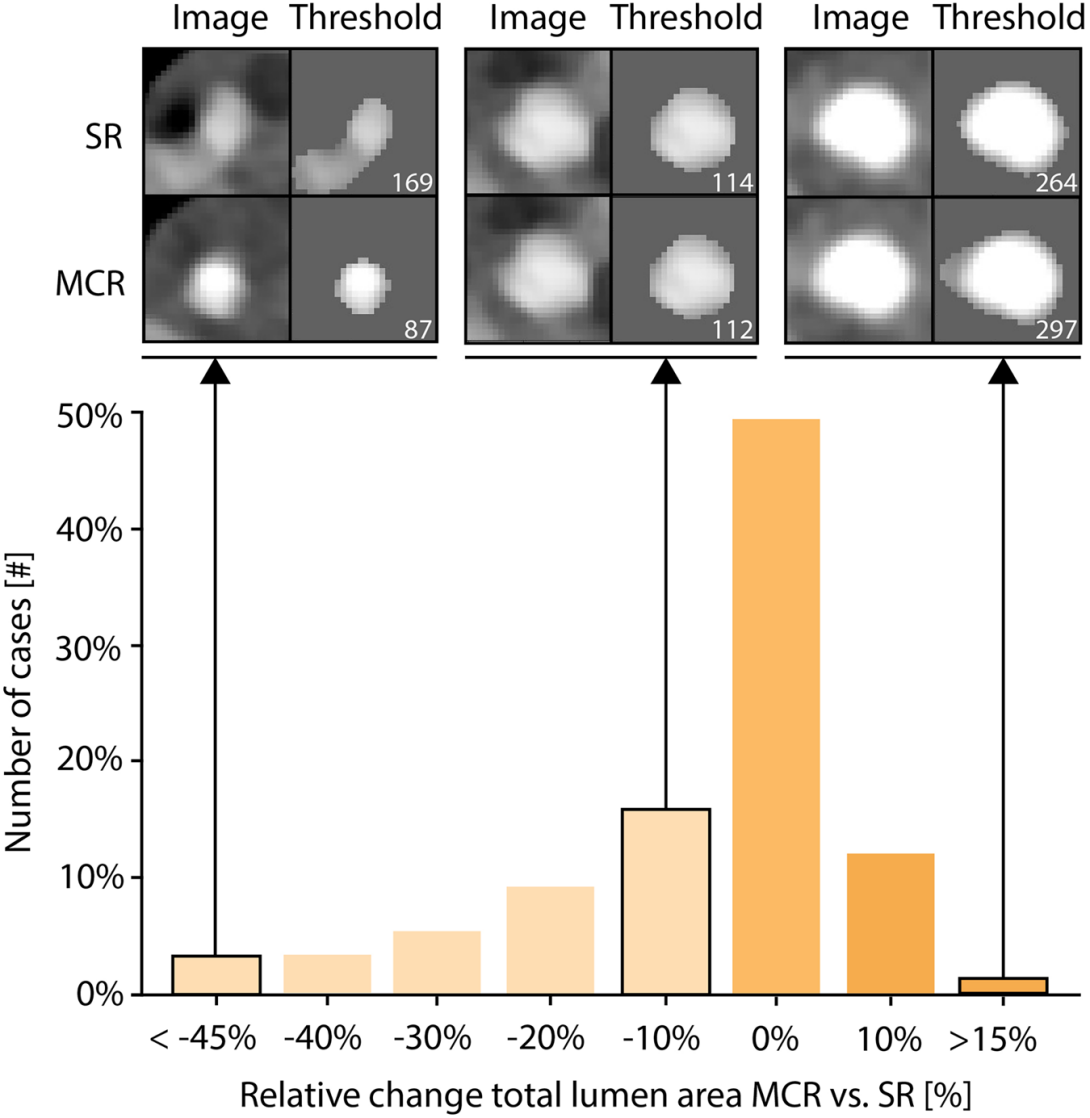
Bar chart of relative motion area with representative images of the standard reconstruction and motion-compensated reconstruction. Bar chart presenting the distribution of cases based on the relative difference in motion area between motion compensated reconstruction (MCR) and standard reconstruction (SR), where 0% represents cases with no difference. The bars to the left indicate a decrease in motion area with MCR compared to SR, while the bars to the right show an increase. Representative images indicating the motion area and segmentation results for SR and MCR are indicated at the top of the figure. The resulting motion area in pixels is given in the bottom-right corner

For all 150 patients, median motion area was 13.4 mm^2^ (10.4–18.2) for SR and 12.6 mm^2^ (10.0–16.5) (*p* < 0.001) for MCR, representing a 6.0% area reduction overall. Figure 2 presents a bar chart illustrating the skewness of the quantified data. Per HR category, motion area (SR vs. MCR) was 12.7 mm^2^ (10.7–16.4) vs. 12.7 mm^2^ (11.0–16.3) (*p* = 0.852) at ≤ 60 bpm, 16.1 mm^2^ (13.4–21.2) vs. 15.3 mm^2^ (12.8–20.4) (*p* = 0.021) at 61–69 bpm, and 16.9 mm^2^ (13.5–25.9) vs. 15.3 mm^2^ (11.8–20.4) (*p* < 0.001) at ≥ 70 bpm.

In the subgroup of 17 patients with a motion artifact as subjectively scored by the readers, median motion area was reduced significantly (*p* < 0.002) with MCR from 14.7 mm^2^ (13.4–18.2) to 12.8 mm^2^ (10.5–15.1), for SR and MCR, respectively. The median difference in motion area was 17%, with a maximum reduction of 54%.

### Subjective image quality

#### All HR

**Fig. 3:**
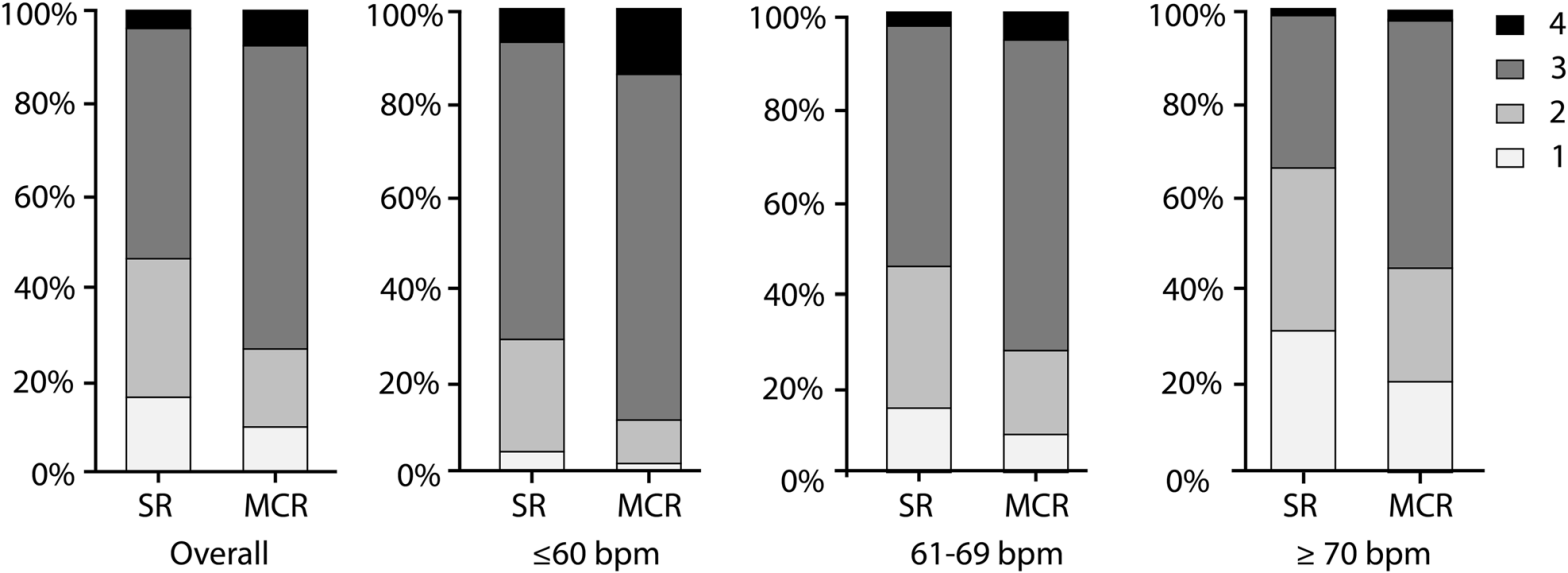
Per-segment subjective image quality of coronary CTA with and without motion compensation. Per segment median subjective image quality (Likert) scores overall and per heart rate category for motion compensated reconstruction (MCR) and standard reconstruction (SR). bpm, beats per minute

Median SIQ scores are presented in Fig. 3 and Table 3. SR SIQ was significantly lower than MCR for all HR combined (*p* < 0.001) on segment (SR: 3.0 (2.0–3.0) vs. MCR: 3.0 (2.0–3.0)), vessel (2.8 (2.0–3.0) vs. 3.0 (2.7–3.0)), and patient levels (2.0 (1.0–3.0) vs. 3.0 (1.0–3.0)). Per-segment SIQ interobserver agreement was 0.80 for SR and 0.77 for MCR. In 11 cases, MCR introduced erroneous corrections to the images that distorted and blurred the coronary artery in part of a segment, thus reducing IQ.

**Table 3:** Subjective image quality scores of coronary CTA with and without motion compensation

	All heart rates			≤ 60 bpm			61–69 bpm			≥ 70 bpm		
	SR	MCR	*p*-value	SR	MCR	*p*-value	SR	MCR	*p*-value	SR	MCR	*p*-value
Per segment	3.0 (2.0–3.0)	3.0 (2.0–3.0)	< 0.001^a^	3.0 (2.0–3.0)	3.0 (3.0–3.0)	< 0.001^a^	3.0 (2.0–3.0)	3.0 (2.0–3.0)	< 0.001^a^	2.0 (1.0–3.0)	3.0 (2.0–3.0)	< 0.001^a^
Per vessel												
All	2.8 (2.0–3.0)	3.0 (2.7–3.0)	< 0.001^a^	3.0 (2.7–3.0)	3.0 (3.0–3.0	< 0.001^a^	2.8 (2.3–3.0)	3.0 (2.7–3.0)	< 0.001^a^	2.3 (1.7–3.0)	3.0 (2.0–3.0)	< 0.001^a^
RCA	2.3 (2.0–2.8)	2.7 (2.0–3.0)	< 0.001^a^	2.7 (2.5–3.0)	3.0 (2.7–3.1)	0.026^a^	2.3 (2.0–2.7)	2.7 (2.3–3.0)	0.002^a^	2.0 (1.5–2.3)	2.0 (1.3–2.7)	0.462
LM	3.0 (3.0–3.0)	3.0 (3.0–3.0)	0.002^a^	3.0 (3.0–3.0)	3.0 (3.0–4.0)	0.004^a^	3.0 (3.0–3.0)	3.0 (3.0–3.0)	0.74	3.0 (2.0–3.0)	3.0 (3.0–3.0)	0.03^a^
LAD	2.7 (2.3–3.0)	3.0 (2.7–3.0)	< 0.001^a^	3.0 (2.7–3.0)	3.0 (3.0–3.0)	0.002^a^	2.7 (2.3–3.0)	3.0 (2.7–3.0)	0.003^a^	2.3 (1.7–2.8)	3.0 (2.4–3.0)	< 0.001^a^
LCx	2.5 (2.0–3.0)	3.0 (2.5–3.0)	< 0.001^a^	3.0 (2.5–3.0)	3.0 (3.0–3.0)	< 0.001^a^	2.5 (2.0–3.0)	3.0 (2.9–3.0)	< 0.001^a^	2.0 (1.5–3.0)	2.8 (2.3–3.0)	0.004^a^
Per patient	2.0 (1.0–3.0)	3.0 (1.0–3.0)	< 0.001^a^	3.0 (2.0–3.0)	3.0 (3.0–3.0)	< 0.001^a^	2.0 (1.0–3.0)	3.0 (1.8–3.0)	0.002^a^	1.0 (1.0–2.0)	1.0 (1.0–2.5)	0.22

*bpm* beats per minute, *MCR* motion compensated reconstruction, *SR* standard reconstruction

^a^ Significant *p*-values

#### Per HR category

For HR categories ≤ 60 bpm and 61–69 bpm, SIQ differed significantly between SR and MCR at all levels (*p* ≤ 0.002) (Table 3). At ≥ 70 bpm, SR SIQ was significantly lower than MCR on a segment and vessel level (*p* < 0.001), but not on a per-patient level.

### Diagnostic interpretability

#### All HR

Overall, diagnostic interpretability improved significantly with the use of MCR (*p* < 0.002) on a segment (1706/1970 (86.7%) vs. 1814/1970 (92.1%)), vessel (509/600 (84.8%) vs. 540/600 (90.0%)), and patient level (81/150 (54.0%) vs. 103/150 (68.7%)) for SR and MCR, respectively (Table 4).

**Table 4:** Diagnostic interpretability of coronary CTA with and without motion compensation

	All heart rates			≤ 60 bpm			61–69 bpm			≥ 70 bpm			
	SR	MCR	*p*-value	SR	MCR	*p*-value	SR	MCR	*p*-value	SR		MCR	*p*-value
Per segment	1706/1970 (87%)	1814/1970 (92%)	< 0.001^a^	653/668 (98%)	661/668 (99%)	0.077	576/649 (89%)	605/649 (93%)	< 0.001^a^	485/653 (72%)		547/653 (84%)	< 0.001^a^
Per vessel													
All	509/600 (85%)	540/600 (90%)	< 0.002^a^	194/200 (97%)	197/200 (99%)	0.453	175/200 (88%)	183/200 (92%)	0.115	140/200 (70%)		156/200 (78%)	0.027^a^
RCA	106/150 (71%)	110/150 (73%)	0.556	48/50 (96%)	48/50 (96%)	1	34/50 (68%)	40/50 (80%)	0.109	24/50 (48%)		22/50 (44%)	0.791
LM	148/150 (99%)	149/150 (99%)	1	50/50 (100%)	50/50 (100%)	1	50/50 (100%)	50/50 (100%)	1	48/50 (96%)		49/50 (98%)	1
LAD	131/150 (87%)	144/150 (96%)	0.007^a^	49/50 (98%)	49/50 (98%)	1	46/50 (92%)	49/50 (98%)	0.375	36/50 (72%)		46/50 (92%)	0.013^a^
LCx	125/150 (84%)	134/150 (89%)	0.093	47/50 (94%)	50/50 (100%)	0.25	45/50 (90%)	44/50 (88%)	1	33/50 (66%)		40/50 (80%)	0.118
Per patient	81/150 (54%)	103/150 (69%)	< 0.002^a^	39/50 (78%)	46/50 (92%)	0.065	27/50 (54%)	38/50 (76%)	0.013^a^	15/50 (30%)		19/50 (38%)	0.454

*bpm* beats per minute, *MCR* motion compensated reconstruction, *SR* standard reconstruction

^a^ Significant *p*-values

#### Per HR category

At ≤ 60 bpm, MCR did not significantly improve diagnostic interpretability at any assessed level (*p* > 0.065), as expected. However, at increased HR of 61–69 and ≥ 70 bpm, diagnostic interpretability was significantly higher with MCR than with SR at all levels (*p* ≤ 0.027), except on a per-vessel level at 61–69 bpm (*p* = 0.115) and on a patient level at ≥ 70 bpm (*p* = 0.454). Regardless, MCR showed a trend of improved interpretability at all HR and assessed levels. Most motion artifacts were reported in the RCA (SR: 16/25 (64%) and MCR: 10/25 (40%)).

### Individual coronary segment assessment at high HR

**Fig. 4:**
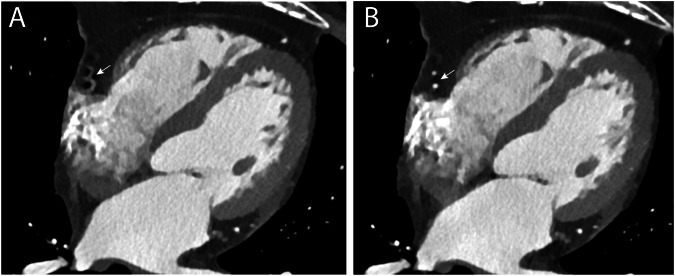
Standard reconstruction vs. motion compensation reconstruction of the coronary CTA image. Clinical examples of a standard coronary computed tomography angiography image (physiologic cardiac phase 78% RR interval) that (**A**) contains severe motion artifacts in the right coronary artery (non-diagnostic segment, white arrowhead) with standard reconstruction (SR) and (**B**) with motion compensated reconstruction (MCR) that reduced the motion artifact, enabling accurate assessment of this segment (Likert score 3)

**Fig. 5:**
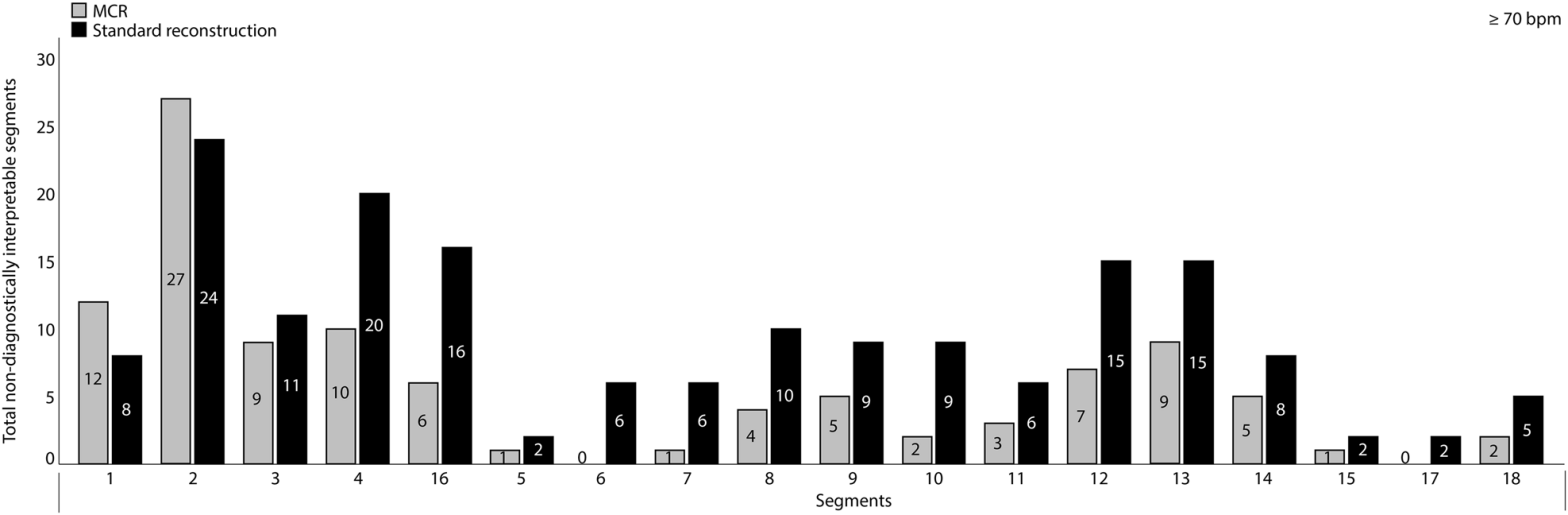
Histogram of the total number of non-diagnostic segments at ≥ 70 bpm of coronary CTA with and without motion compensation. Total number of non-diagnostic segments (Likert score of 1) across 50 patients at ≥ 70 beats per minute (bpm) is shown for motion compensated reconstruction (MCR) (gray) and standard reconstruction (SR) (black) at 78% of the cardiac phase. Each bar represents the number of non-diagnostic scores per respective segment. For all segments, the corresponding number of cases is given centrally in each bar

In the ≥ 70 bpm category, segment 2 of the RCA had the most non-diagnostic scores for both SR and MCR (Figs. 4 and 5). Overall, MCR decreased the amount of non-diagnostic scores by 40%, with the largest reduction in segment 4 (50%) and segment 16 (63%), salvaging 10 cases in each segment. MCR showed lower non-diagnostic scores in all high HR segments, except for segments 1 and 2, see Fig. 5.

### Multi-phase comparison

Across all patients, MCR was ranked as the best or equally best overall SIQ in 72% (105/146) of the cases compared to all available phases (76%, 78%, 80%, and MCR). In 12% of cases (18/146), however, MCR was not the optimal choice, see Appendix 2. In the subset where the target phase (78%) was scored as suboptimal and a multi-phase assessment would clinically be preferred, MCR was the single best alternative in 76% (44/58) of cases. Notably, in 31% (18/58), MCR was the only reconstruction that provided a diagnostically interpretable image. In 24% (14/58), MCR was not the preferred reconstruction.

## Discussion

This retrospective clinical CCTA study assessed the impact of a vendor-specific MCR on DLCT IQ and diagnostic interpretability across multiple HR. Our key findings demonstrated that (A) MCR significantly improved SIQ per-segment, per-vessel, and per-patient, as well as diagnostic interpretability across most HR categories compared to the SR at 78% of the RR-interval, (B) thereby avoiding the necessity of assessing additional cardiac phases, and (C) significantly improved the OIQ by reducing motion artifact areas.

Accurate coronary artery assessment in CCTA is often complicated by cardiac movement during image acquisition, causing motion artifacts and non-diagnostic segments [8]. In this study, MCR with DLCT CCTA significantly reduced non-diagnostic coronary segments at higher HR, reflected in fewer non-diagnostic scans (per-patient scores) at 61–69 bpm. As part of a post hoc analysis, we evaluated all available phases (75, 76%, 78%, 80%, and MCR) in a blinded fashion and ranked them according to overall image quality and diagnostic interpretability. When the 78% phase is suboptimal, MCR is most often the single best (76%) alternative and serves as the only diagnostic reconstruction available in 31% of cases, thereby salvaging scans that would otherwise have been uninterpretable. Nevertheless, in a minority of cases, persistent or even worsened artifacts may limit its utility. For this reason, although MCR may provide a convenient first step when a non-diagnostic segment is identified on the 78% RR-interval, we still recommend that all phases and MCR should be reconstructed by default to facilitate multi-phase assessment when necessary. Particularly, salvaging CCTA images in this HR category (61–69 bpm) is important, as this category typically concerns the group of patients in which preparation (beta-blockers, instructions) yielded unsatisfactory results (i.e., not reaching a target HR of < 60 bpm). Nonetheless, while per-patient SIQ and diagnostic interpretability improved at ≥ 70 bpm, the differences were not statistically significant, likely attributed to strict CAD-RADS 2.0 criteria and the significantly shortened mid/late diastolic and diastasis rest phase [8]. One non-interpretable segment rendered the corresponding vessel and patient non-diagnostic (Likert 1). At ≤ 60 bpm, diagnostic interpretability improved with MCR, but not significantly, possibly as motion artifacts in general are less common at lower HR.

To objectively assess MCR’s impact on reducing these artifacts, we integrated a novel and comprehensive technique to objectively measure motion areas. With this, our previous phantom study reported an 11-fold reduction in the area of motion artifacts with MCR [25]. In the current study, we validated these results, showing significant motion artifact area reduction in patients, although less pronounced. It is important to consider that complex physiological 3D movement, individual anatomical and tissue differences in patients, and specific HU thresholds to differentiate between lumen and non-lumen will influence segmentation performance, which was not a factor in the controlled phantom environment.

This study’s MCR is an advanced, fully automated tool that acts during acquisition and back projection, and thus is not a post-processing tool. Its exact function and results, therefore, cannot directly be compared to those of other vendors. A recent clinical study using the same approach by Liu et al was published at the time of writing and included 127 patients undergoing CCTA with suspected CAD, divided into two HR groups (< 75 bpm and ≥ 75 bpm) [30]. MCR significantly improved Likert scores and interpretability at the segment, vessel, and patient levels across both HR groups, except for the interpretability of the LAD and Cx at < 75 bpm. MCR also significantly improved signal-to-noise ratio (SNR) and contrast-to-noise ratio (CNR). However, the authors only used two broad HR groups, utilized retrospective gating, and based Likert scores solely on motion artifacts, excluding other IQ parameters.

Although we are the first to study OIQ as presented, directly comparing our clinical results to previous CCTA motion correction studies is challenging and perhaps less relevant. This is due to not only different motion correction techniques, but also differences in populations, CT systems and generations, and acquisition and reconstruction protocols. Using a different vendor, Fuchs et. al (2014) showed that their first generation of image-based motion correction significantly improved per-segment and vessel IQ and interpretability in patients with HR < 63 bpm (*n* = 40), despite the lack of further subgroup analyses [21]. Similarly, Machida et al (2014) found significant per-segment and vessel improvements in SIQ and interpretability across multiple HR categories with the same approach, although their study focused only on motion artifacts and used mixed gating approaches, complicating direct HR comparisons [31]. Sheta et al (2017) performed a randomized controlled trial comparing the same motion correction to SR in patients randomized to receive beta-blockers before CCTA, concluding that motion correction improves IQ and reduces motion artifacts, but cannot entirely replace beta-blockers [32]. The second generation of their motion correction has demonstrated improved IQ over the first generation and SR, even at high HR in both adult and pediatric patients [33–36]. Looking forward, photon-counting CT may provide higher spatial resolution, thereby improving SIQ and diagnostic interpretability beyond current spectral systems [37]. Integrating photon-counting CT with existing MCR techniques could further enhance CCTA at high HR. Whether this may allow for CCTA without beta blocker preparation requires further evaluation. The growing need for high-quality single-phase acquisitions in CT-fractional flow reserve-guided treatment and PCI planning highlights the relevance of MCR. Accurate blood flow simulation and reliable stenosis assessment require high-quality images, and motion artifacts in suboptimal CCTAs can reduce accuracy or lead to rejection, underscoring the importance of optimizing image reconstructions for improving image quality and diagnostic confidence [38, 39].

Several limitations might have influenced our results. First, OIQ was assessed on a manually selected slice in segment 2 of RCA, which may not fully represent the overall IQ. Second, the OIQ analysis utilized a 120HU threshold for isolating lumen areas compared to 80HU used previously due to differences in surrounding tissue composition between the phantom (water) and actual patients. This needed change affects segmentation performance and complicates direct comparisons. Third, MCR caused a coronary segment downgrade that distorted and blurred the coronary artery in part of a segment in some instances, typically where there was no appreciable motion. Our current MCR is therefore useful in cases with actual coronary segment motion, for example, when the HR unexpectedly rises during scanning. As MCR should only be assessed when motion artifacts are present, MCR-induced artifacts are unlikely to impede the interpretation of clinical readings. In addition, artifacts do not resemble clinical coronary pathology and can be differentiated. At higher HR (e.g., > 70 bpm), the selected rest phase (diastole) becomes significantly impacted and shortened, and the end-systolic rest phase may be a better target. Despite careful rest phase selection based on initial HR, factors like stress or contrast agents can raise HR during acquisition beyond initially anticipated, underscoring that SR and MCR are both needed. Fourth, given that MCR was primarily only assessed in comparison to a solitary 78% RR-interval phase SR on a per-segment and vessel level, the improvement in diagnostic interpretability may not be fully representative of a CCTA with multiple phases reconstructed. Fifth, our findings are not generalizable to other vendors due to inherent vendor-specific differences. Lastly, the impact of heart rate variability during the scan phase was not assessed, as the full ECG data were not stored systematically for our cohort.

Future research should explore expanding MCR to assess IQ and diagnostic interpretability at even higher HR, including in acute settings where higher HR and non-ECG-triggered scans can be expected. This particularly benefits pediatric patients, who often have naturally higher and more variable HR, making standard imaging techniques challenging. Additionally, evaluating MCR’s effectiveness in scans performed without beta-blockers could help optimize CCTA protocols for patients who cannot tolerate these medications. Moreover, assessing the impact of MCR on the accuracy of coronary stenosis measurements would provide further validation of MCR’s clinical utility. Addressing these areas may further enhance CCTA’s clinical utility, particularly in populations that are currently difficult to image effectively.

## Conclusion

In conclusion, our clinical coronary computed tomography angiography study demonstrated that the applied vendor-specific motion compensated reconstruction (MCR) significantly improves subjective image quality (SIQ) and diagnostic interpretability across most heart rates and in major vessels (diameter ≥ 1.5 mm) in coronary dual-layer spectral CT compared to the SR at 78% of the RR-interval. Hence, by applying this technology, MCR offers benefits in terms of motion artifact reduction, SIQ, and diagnostic interpretability.

## Supplementary information

ELECTRONIC SUPPLEMENTARY MATERIAL

## Abbreviations

bpm: Beats per minute
CAD: Coronary artery disease
CAD-RADS: Coronary Artery Disease-Reporting and Data System
CCTA: Coronary computed tomography angiography
Cx: Circumflex artery
DLCT: Dual-layer computed tomography
ECG: Electrocardiogram
HR: Heart rate
HU: Hounsfield units
IMR: Iterative model reconstruction
IQ: Image quality
kVp: Kilovoltage peak
mAs: Milliampere-seconds
MCR: Motion-compensated reconstruction
OIQ: Objective image quality
RCA: Right coronary artery
SIQ: Subjective image quality
SRs: Standard reconstructions
