# Supplementary material for: Motion compensated reconstruction improves image quality and interpretability of dual-layer coronary CT angiography
Source: Eur Radiol. 2025 Sep 3;36(3):1977–88. doi: 10.1007/s00330-025-11946-x (PMC12963155; doi:10.1007/s00330-025-11946-x)
Supplement: Supplementary file 1 — ELECTRONIC SUPPLEMENTARY MATERIAL [file 330_2025_11946_MOESM1_ESM.pdf]

Motion compensated reconstruction improves image quality and interpretability of dual-layer coronary CT angiography

ELECTRONIC SUPPLEMENTARY MATERIAL

Appendix 1: Flowchart of the study population and analyses

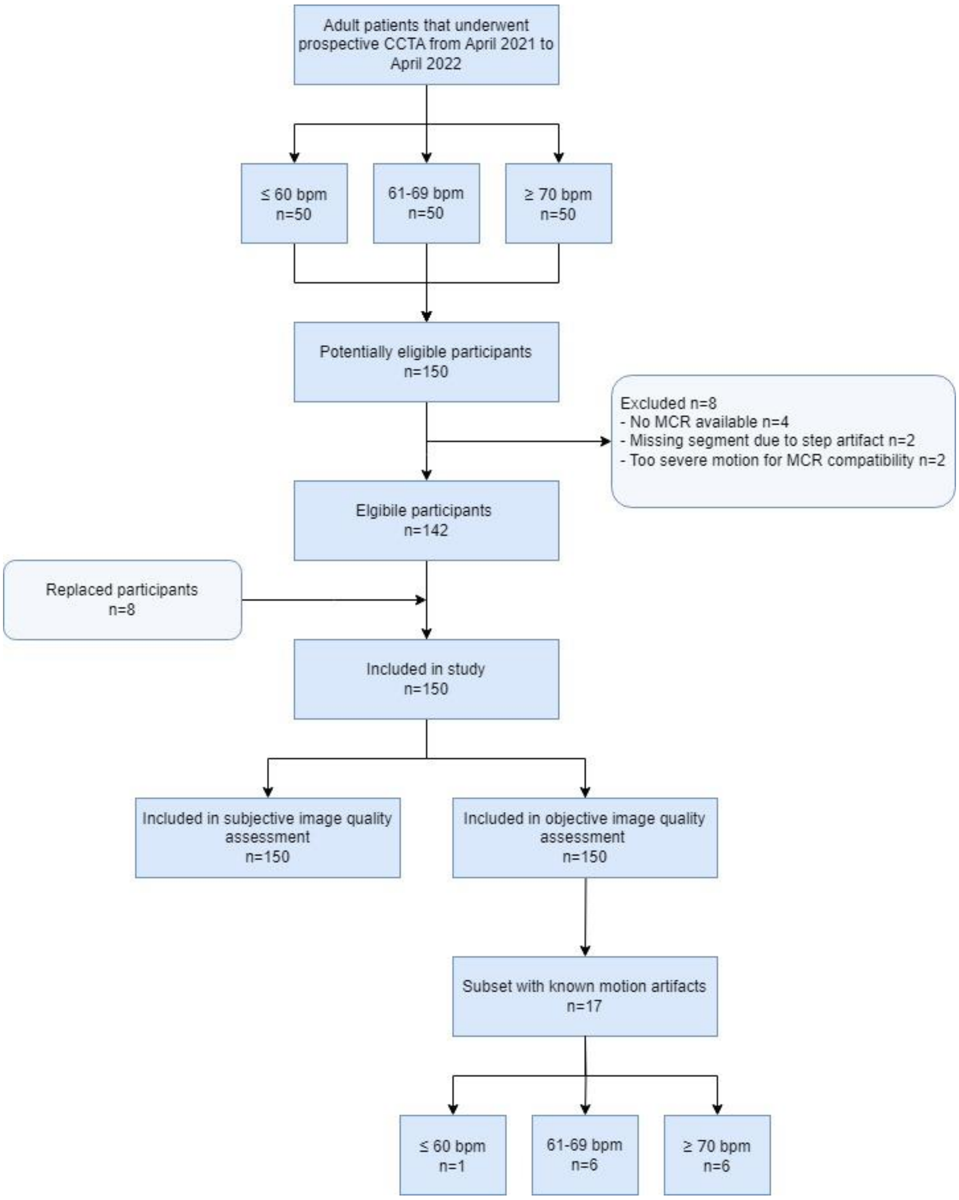

Abbreviations: CCTA: coronary computed tomography angiography; bpm: beats per minute; MCR: motion compensated reconstruction

Appendix 2: Multi phase comparison of motion compensated reconstruction

Categories	n (%)
**Total cases identified**	150 (100%)
**Excluded**	4 (3%)
Missing phases	4
**Included**	146 (97%)
MCR best	45 (31%)
MCR equally best	60 (41%)
MCR intermediate	23 (16%)
MCR worst	18 (12%)
**78% not the best phase**	58 (40%)
MCR best	44 (76%)
MCR equally best	0
MCR intermediate	11 (19%)
MCR worst	3 (5%)
**Only MCR diagnostically interpretable**	18 (31%)

Abbreviations: MCR: motion compensated reconstruction.
